# Frequency and distribution of neglected tropical diseases in Mozambique: a systematic review

**DOI:** 10.1186/s40249-019-0613-x

**Published:** 2019-12-13

**Authors:** Berta Grau-Pujol, Marilia Massangaie, Jorge Cano, Carmen Maroto, Alcino Ndeve, Francisco Saute, Jose Muñoz

**Affiliations:** 1 ISGlobal, Hospital Clínic, Universitat de Barcelona, Barcelona, Spain; 2 Centro de Investigação em Saúde da Manhiça (CISM), Maputo, Mozambique; 3 Mundo Sano Foundation, Buenos Aires, Argentina; 4 Direcção Nacional de Saúde Pública, Ministério da Saúde, Maputo, Mozambique; 5 Faculty of Infectious and Tropical Diseases, London School of Hygiene and Tropical Medicine, London, UK; 6 Liverpool School of Tropical Medicine, Liverpool, UK; 7 No Leprosy Remains, Maputo, Mozambique

**Keywords:** Neglected tropical diseases, Mozambique, Africa, Epidemiology, Poverty, Infectious diseases, Review, Helminth, Trachoma, Leprosy

## Abstract

**Background:**

Neglected tropical diseases (NTDs) affect more than one billion people living in vulnerable conditions. In spite of initiatives recently contributing to fill NTDs gaps on national and local prevalence and distribution, more epidemiological data are still needed for effective control and elimination interventions.

**Main text:**

Mozambique is considered one of the countries with highest NTDs burden although available data is scarce. This study aims to conduct a systematic review on published available data about the burden and distribution of the different NTDs across Mozambique since January 1950 until December 2018. We identified manuscripts from electronic databases (Pubmed, EmBase and Global Health) and paper publications and grey literature from Mozambique Ministry of Health. Manuscripts fulfilling inclusion criteria were: cross-sectional studies, ecological studies, cohorts, reports, systematic reviews, and narrative reviews capturing epidemiological information of endemic NTDs in Mozambique. Case-control studies, letters to editor, case reports and case series of imported cases were excluded. A total of 466 manuscripts were initially identified and 98 were finally included after the revision following PRISMA guidelines. Eleven NTDs were reported in Mozambique during the study span. Northern provinces (Nampula, Cabo Delgado, Niassa, Tete and Zambezia) and Maputo province had the higher number of NTDs detected. Every disease had their own report profile: while schistosomiasis have been continuously reported since 1952 until nowadays, onchocerciasis and cysticercosis last available data is from 2007 and Echinococcosis have never been evaluated in the country. Thus, both space and time gaps on NTDs epidemiology have been identified.

**Conclusions:**

This review assembles NTDs burden and distribution in Mozambique. Thus, contributes to the understanding of NTDs epidemiology in Mozambique and highlights knowledge gaps. Hence, the study provides key elements to progress towards the control and interruption of transmission of these diseases in the country.

## Multilingual abstracts

Please see Additional file 1 for translations of the abstract into the five official working languages of the United Nations.

## Background

Neglected tropical diseases (NTDs) are a group of diseases mainly caused by a virus, bacterium, protozoon or helminth. Even though they have distinct biological and transmission features, they all affect the most vulnerable populations, primarily in low socio-economic countries — one billion people annually [1]. NTDs lead to elevated morbidity, child-maternal health complications and impaired child development. Hence, they affect the quality of life and contribute to a cycle of poverty in endemic populations [2, 3].

NTDs are targeted in the context of the Sustainable Development Goal (SDG) 3.3: “To end the epidemics of Acquired Immunodeficiency Syndrome (AIDS), tuberculosis, malaria, NTDs, hepatitis, water-borne diseases and other communicable diseases by 2030.” [4] Unfortunately, the scarcity of epidemiological data for NTDs hampers the implementation of control and elimination activities in many endemic countries [5]. Epidemiological information about the presence of NTDs, including spatial mapping, is key for effective implementation of interventions [2]. In spite of public and private initiatives recently contributing to fill NTDs gaps on national and local prevalence and distribution, more epidemiological data are still needed for effective control and elimination interventions.

Mozambique is a country in sub-Saharan Africa that consists of an area of 786 000 km^2^ with a population of 29 million [6], 46.1% of them living with poverty conditions [7]. Although Mozambique is one of the countries with the highest total burden of NTDs [8, 9], very limited data about the burden and distribution of these diseases is available [1].

A comprehensive and systematic review of scientific literature on NTDs is a first step to understand where these diseases may be present and what populations are more exposed in the country. This will allow us to identify existing gaps in disease mapping. Thus, we aim at reviewing and assembling published available data about the prevalence and distribution of the different NTDs across Mozambique since 1950. The outcome could guide future Mozambican and international research, and will also support and influence future health policy decisions in this country which may be extrapolated worldwide.

## Main text

### Methods

#### Search strategy

This review is based on the Preferred Reporting Items for Systematic Reviews and Meta-Analysis (PRISMA) guidelines (Additional files 2 and 3). We conducted an electronic literature search using EndNote X7.8 throughout the following electronic databases: Pubmed/MEDLINE, EmBase and Global Health. We did not apply restrictions to language of publication. The results were limited in the search string to articles published from January 1950 to December 2018. Five NTDs recently considered by the World Health Organization (WHO) (chikungunya, mycetoma, chromoblastomycosis, scabies and snakebite) were not included. Chagas was not included in the search due to its geographical distribution limited to Latin America. The search terms used are found in Additional file 4. We conducted additional search on paper publications and grey literature (conference proceedings, abstracts, masters and doctoral theses) available in Mozambique Ministry of Health library. We also extended the search to the reference lists of ascertained articles to identify more publications that met searching criteria. To be eligible for inclusion, studies had to be cross-sectional studies, ecological studies, cohorts, reports, systematic reviews, and narrative reviews capturing information related to epidemiology of endemic NTDs in Mozambique. Case reports and case series were considered for inclusion if studies confirmed national case location and they were not imported cases. We excluded case-control studies, letters to editor and case reports and case series of imported cases. Studies with participants based out of the country of Mozambique and studies based on mathematical modelling of epidemiological data were also excluded. No restrictions were made with diagnosis strategy.

#### Study selection

**Fig. 1:**
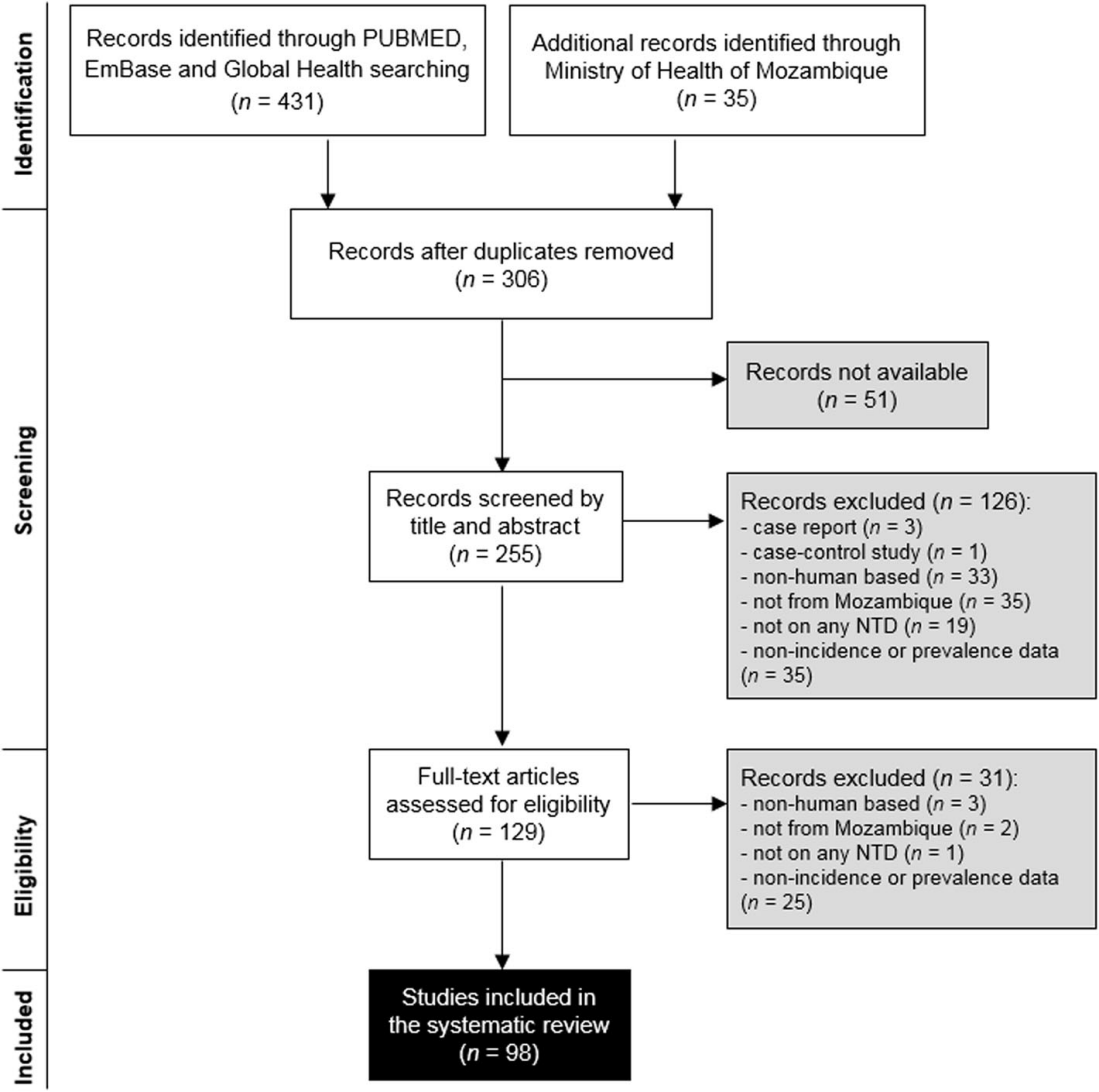
Selection of the sources included in the review

The methods used in selecting manuscripts are provided in Fig. 1.

### Results

#### Demographic trends

**Fig. 2:**
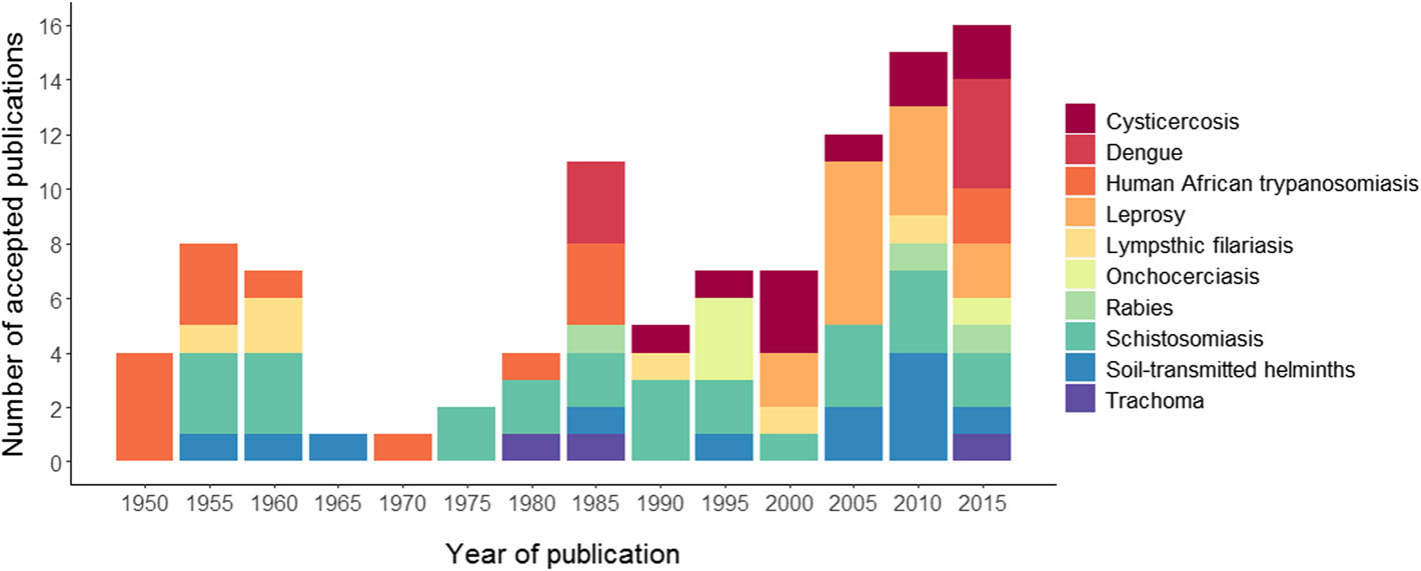
Number of NTDs publications in Mozambique during 1950–2018 accepted by full-text, displayed every five years. NTDs: Neglected tropical diseases

Figure 2 illustrates the trends in publications accomplishing the inclusion criteria. Until 2004, the number of articles that provide information on the prevalence or incidence of NTDs in Mozambique was quite low, with a minimum and a maximum of one article and 11 articles in 5 years respectively. The period with lower number of publications coincide with the Mozambican Independence War (1964–1974). The last 13 years, this numbers had steadily increased. In addition, from 2015 onwards, a higher variety of NTDs are studied in the country coinciding with the international recognition of NTDs as a public health priority in low and middle income countries, as was highlighted in the London Declaration of NTD in 2012. The publication trend for each NTD between 1950 and 2018 has its own character, while we can find studies on Human African trypanosomiasis in 1952 and the last published about it was in 1987, no studies on cysticercosis are found until 1990. Conversely, we found schistosomiasis studies from 1957 until nowadays.

**Fig. 3:**
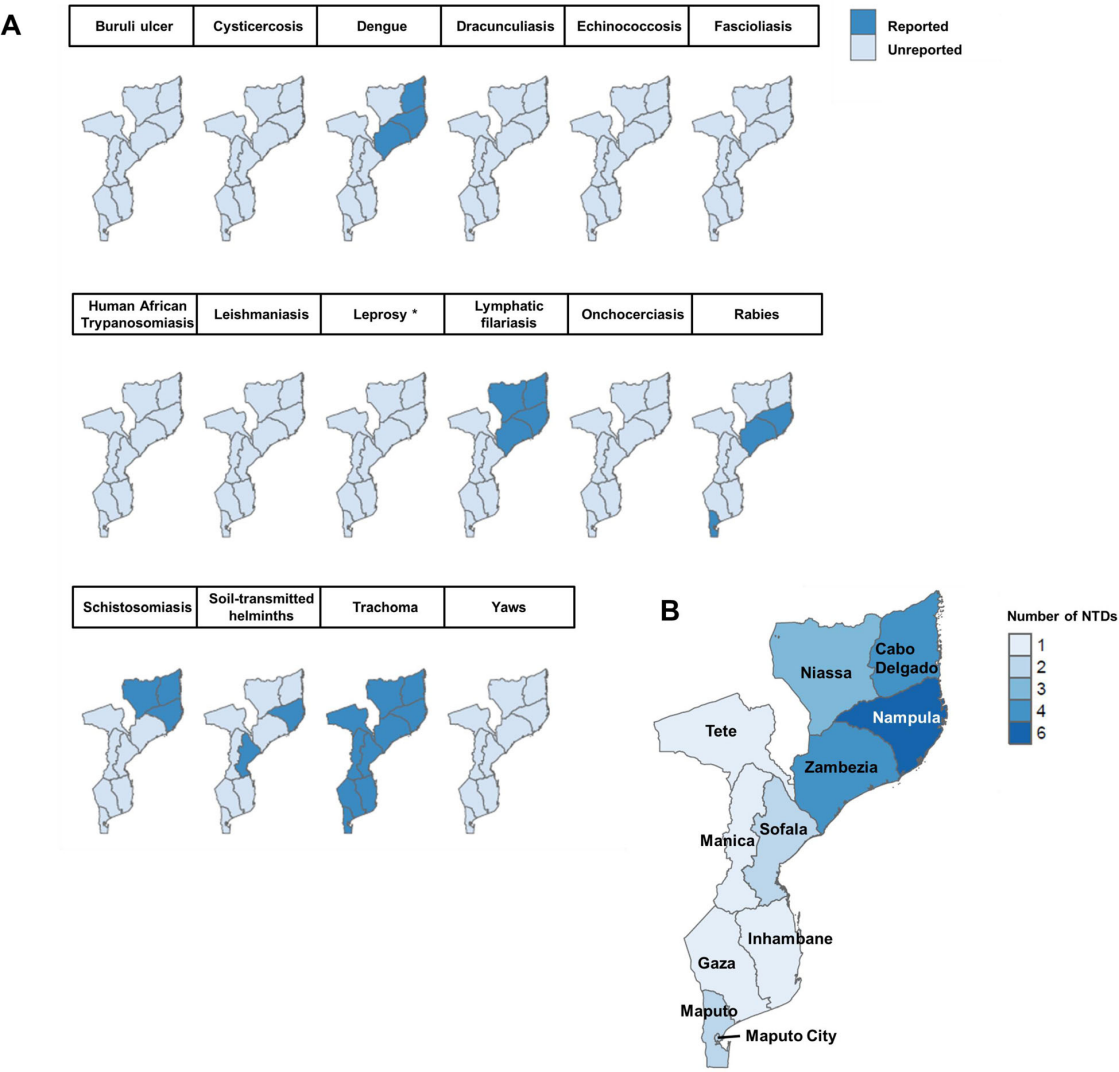
NTDs distribution in Mozambique per province. **a**, provincial map for each specific NTD identified per province during 2008–2018 in Mozambique, **b**, number of NTDs identified per province during 2008–2018 in Mozambique. These maps are original, build with R (http://www.R-project.org/). Cartographic resources were obtained from GADM (www.gadm.org), epidemiological data was obtained from the manuscripts reviewed. See Additional file 5. NTDs: Neglected tropical diseases. *Only national data but not provincial data is provided

Considering the data collection time, the Northern provinces (Nampula, Cabo Delgado, Niassa and Tete), Zambezia and Maputo and Maputo city had the highest number of NTDs idenified along the study span. For the last 10 years, Gaza, Manica and Inhambane are the provinces with less NTDs reported. During this period, viral NTDs have been observed in the north of the country and Maputo province, helminth’s NTDs in the northern provinces, bacterial NTDs in all of them while protozoan NTDs were not reported. Figure 3 illustrates the reported distribution of NTDs per province in Mozambique during the last 10 years. The information shown in the map is limited by the few publications available per disease.

#### Neglected viral infections

##### Dengue fever

The first ever documented case of dengue virus transmission was caused by a serotype 3 in Pemba Province in October 1984. After a peak in December 1984, the outbreak concluded in March 1985. Although there is limited information regarding the extent of the epidemic, it is estimated to have affected 45% of Pemba province population, with no observable differences in gender, age or neighborhood [10–12]. Cases were confirmed using hemagglutination-inhibition (HI), complement fixation (CF) and IgM antibody capture enzyme-linked immunosorbent assay (ELISA) [13]. In 2014, thirty years after the first documented outbreak, another dengue outbreak occurred in Pemba and Nampula. A total of 193 clinically suspected dengue cases were detected, of those three were detected with reverse transcriptase polymerase chain reaction (PCR) in sera and 97 tested positive for dengue virus (DENV) NS1 antigen or anti-DENV IgM antibody. No further cases of dengue have been reported in Mozambique after this outbreak.

##### Rabies

The number of human rabies cases notified decreased from 48 in 1978 to five in 1981 with a mild increase in 1982. This tendency was suggested to be related to animal vaccination, which followed the opposite trend. From 1978 until 1982, the mean annual incidence of rabies in Mozambique was 0.2/100000 inhabitants with a total of 123 cases registered. Tete was the province with more human rabies cases notified (22 cases) and Niassa with fewer cases (four cases) [14]. Few years later, the number of human rabies notifications sharply increased in the country [15]. The annual average of cases from 2008 until 2010 was 40.3, and according to the World Animal Health Information System 72 cases occurred in 2011. Maputo province had the highest human rabies case number per inhabitant, followed by Zambezia and Nampula provinces. During this period, case confirmation was conducted with fluorescent antibody test (FAT) at Central Veterinary Laboratory [15]. In 2014, there was an outbreak of 14 cases in Maputo and Matola cities and Boane neighbouring district. All cases were clinically detected with no laboratory confirmation, they were recently bitten by a dog and they were admitted in Maputo Central Hospital [16].

#### Neglected bacterial infections

##### Trachoma

Paulo et al. (1984) [17] conducted a trachoma survey by clinical examination in both schoolchildren and community population in a village in Mueda, northern Mozambique. The overall prevalence in schoolchildren was 34%. The prevalence in the community, during clinical visits for eye claims in two neighbourhoods of the village was 67 and 50%. Cairncross and Cliff (1987) [18] conducted a study in two villages in the same area. One was water supplied and observed a trachoma prevalences of 19%, and the other was non-water supplied, with 38% trachoma prevalence. In both villages, most positive cases were adults (67%) and they detected some clustering of cases by household. Next epidemiological data available on trachoma in Mozambique is not until 2012–2015, when Abdala et al. (2017) evaluate 137 districts. They observed a trachomatous inflammation-follicular prevalence > 10% in children 1–9 years old in 20 districts and a trichiasis prevalence ≥0.2% in > 15 years old in 34 districts [19].

##### Leprosy

From 1996 to 1998 there was a decline on new leprosy cases passively identified. However, in 1998, Mozambique was still the sixth country most affected by leprosy in the world with 2412 cases detected (3.4 cases per 10 000 people) [20]. After the implementation of active case detection in 1999, the number of new cases detected increased by 57% (3791 new cases). Niassa, Cabo Delgado and Nampula had the highest prevalence. Concretely, Nampula province, with 19% of the country population, had 55% of leprosy cases. While passive case finding at clinics attracted a higher proportion of women, active case finding significantly detected younger patients (< 15 years old) and paucibacillary cases: patients with less than six patches with clear loss of sensitivity and/or one enlarged peripheral nerve [20, 21]. The annual number of new leprosy cases at a national level in 2003 was 5907, which decreased until achieving the elimination goal at the beginning of 2008. Nevertheless, at that time Mozambique was still among the 17 countries that accounted for 94% of the new cases detected globally, with ≥1000 new cases per country. In 2010 there was small increase in the cases detected (1207 new cases) compared to 2009 (1191 new cases). Following this number, in 2012, the cases decreased to 758. After a 2 year of no cases reporting, in 2015 cases raised again to 1335 in 2015 and to 1926 in 2017, with 243 and 293 new cases identified with grade-2 disabilities (28 of them where children) [22–33].

#### Neglected protozoal infections

##### Human African trypanosomiasis

In Mozambique, only *Trypanosoma brucei rhodesiense* causes human African trypanosomiasis (HAT). The first case in the country was identified in Tete in 1909, confirmed by microscopy [34, 35]. .In 1945, it was created the Mission to Combat Trypanossomiasis (MCT) and active case finding was implemented. That year, 180 cases were identified in the north and northeast of the country. During 1946 and 1948 the Metangula epidemic occurred in Niassa, with 654 cases detected, a mean of 218 cases per year. After that, the number of national cases decreased to 184 in 1949. However, another epidemic occurred during 1952 to 1954 in Mocímbua da Praia, Cabo Delgado, with 705 cases detected. The MCT responded with a chemoprophylactic campaign in that region, decreasing the endemic index to 0.17 cases per 1000 inhabitants in 1956, compared to 0.4 cases per 1000 inhabitants in 1954. Thus, 1956 was the year with fewer cases (127 cases) since 1945: 89 cases in Cabo Delgado, 30 cases in Tete and eight cases in Nampula. However, in 1957, there was an increase of 94 annual cases detected (221 cases detected), with 141 cases in Cabo Delgado, 52 cases in Tete, 27 cases in Nampula and one case in Niassa [35–42]. .Nevertheless, there was 70% decline in 1959, with 63 cases, maintained with a mean of 46 the following 20 years. During 1975–1984, after the Mozambican War of Independence (1964–1974), a total of 739 cases were identified (87% of cases in Tete), and from 1982 onwards there was an increasing number in Niassa and Cabo Delgado [35, 43]. The prevalence of the disease was always reported higher in adult men. All suspicious cases had at least one of these symptoms: fever, adenopathy, oedema and loss of weight. In this way, all cases reported from 1953 onwards, were clinically identified and confirmed microscopically with blood smear or thick blood smear and CSF. Eighty per cent of the patients were diagnosed in the second phase of the disease (parasite invaded the central nervous system) and they were identified by trypanosomes in CSF or CSF with more than 25 mg of proteins/100 ml liquor. Regarding treatment, 4% of the patients in Mozambique were identified as resistant to melarsoprol [43]. More recent, in 2002 and 2004 a case each year was reported in the context of WHO network for HAT elimination, but no cases have been reported later on [44].

#### Helminths infections

##### Onchocerciasis

The situation of onchocerciasis in Mozambique was unknown until 1996, when the disease was confirmed in Zambezia and Tete. In 1998, Mozambique Ministry of Health in conjunction with the World Health Organization selected 60 villages from Cabo Delgado, Niassa, Tete and Zambezia (provinces closer to the borders with endemic countries) with high presence of risk factors for onchocerciasis: (i) close proximity to rivers and rapids, (ii) isolated village and (iii) first river line communities. Thirty to fifty people from the community older than 20 years old with agricultural activities were selected for screening through nodule inspection. For communities bigger than 800 people they invited to participate the population closer to the river. Thus, 3126 people were enrolled: 1769 men and 1357 women. The prevalence of the four provinces was under 2%, with higher prevalence for men and for Niassa and Zambezia provinces. No other provinces were examined [45–47]. In 2001, a Rapid Epidemiological Mapping of Onchocerciasis (REMO) [48] survey in the north of the country observed 50 positive cases from Inhambane, Tete, Niassa, Zambezia or Cabo Delgado. In 2007, another REMO survey observed 61 positive cases from Niassa, Cabo Delgado, Zambezia or Tete. Manica, Sofala and Nampula were also surveyed in 2001 but no cases were found and they were not examined in 2007.

##### Lymphatic filariasis

No cases of lymphatic filariasis (LF) were identified in Mozambique until the campaign of the Mission to Combat Trypanosomiais did in 1953. They detected 14 cases (0.26% of prevalence) of *Wuchereria brancrofti* infection in blood in the human African trypanosomiasis endemic area (Tete, Cabo Delgado, Niassa and Nampula) [49]. During 1959–1960, Nampula showed a prevalence of *W. brancrofti* infection of 5%, Cabo Delgado 6.7%, Manica and Sofala 10.9%, Tete 19.3% and Zambezia 5.4% (1959) and 8% (1960) [50]. Concretely, 87 of adult cases and 21 child cases of *W.brancrofti* infection were identified from 1420 people examined in Zambeze valley during that period [51]. No more data was available until 1990, when a study conducted in Pemba provincial hospital showed a prevalence of infection of 12%, with 8.1% in people from Pemba city and 19.7% in people living outside the city. Although a higher number of women were examined (57%), men represented 63% of the cases. In addition, prevalence of infection in men showed to increase significantly with age, with higher prevalence in men between 21 and 30 years old [52]. In 1998, REMO campaign also benefit the identification of LF cases: they identified 21 cases of hydrocele and four cases of lymphedema in Cabo Delgado, Tete and Zambezia. During the period 1995–2000, hospital histories in Pemba (Cabo Delgado) showed 1300 cases identified, which 99.5% of those had hydroceles and the only cases of lymphedema identified were women. In addition, there was a lower frequency of cases in people < 15 years old [53]. In 2009, Mozambique was still one of the 39 African countries that are endemic for LF, with 16 million people estimated at risk and two million infected; Cabo Delgado and Nampula were the provinces with higher risk [54].

##### Dracunculiasis

All endemic countries for dracunculiasis are in sub-Saharan Africa. Nevertheless, the International Comission for the Certification of Dracunculiasis Eradication held in 2007 certified Mozambique as a country free of transmission [55].

##### Cysticercosis

There is limited available data on cysticercosis in Mozambique. An initial cross-sectional study in Tete district showed absence of *Taenia* infection examined microscopically by Ritchie-Willies technique. But in 1968, the first reported post-mortem neurocysticercosis case was incidentally detected, in association with rabies. Few years later, in 1999, the first clinical neurocysticercosis case was reported in Maputo Central Hospital [56, 57]. Few cross-sectional seroepidemiology studies showed prevalences of 20% in 1994 and 15% in 2009 in Tete [56–58], and 12.1% in 1999 and 20.8% in 2003 in Maputo [57, 58]. Only one case was detected by faecal examination in Maputo in 2003 [57]. In Angonia district, Tete, 51% of the seropositive people of the district presented neurocysticercosis in 2007 and neurocysticercosis-associated epilepsy and headache was present in 5% of the population [59–61].

##### Schistosomiasis

The first case of urinary schistosomiasis was reported in Nampula in 1904 as “tropical haematuria” [62, 63]. Nevertheless, the first schistosomiasis national survey was not performed until 1952–1957 by the Institute of Tropical Medicine in Lisbon. *Schistosoma haematobium* was found in all districts and the district rates ranged between 40 and 80%, while the distribution of *S. mansoni* was as extensive but missing in Cabo Delgado [64]. In fact, settlers with less than a year of residence had schistosome parasite rate of 2.6% in 1956 [65]. In 1961, *S. haematobium* prevalence rates under 50% were only reported in the districts of Maputo and Tete, whereas in Niassa it was over 70% and in Zambezia and Nampula over 80% [62, 66]. *S. mansoni* was determined at the same survey during that period but prevalence was moderate-low (from < 10 to 30%) and not detected in 28 of the 80 districts. *S. mansoni* was only over 12% in Inhambane (19%) and Tete (18.2%) [66]. In 1985, a study in primary schools showed a prevalence of 23.4% for *S. haematobium* and 4.4% for *S.mansoni* [67], and the urinary form was always found far higher than intestinal schistosomiasis [62]. Taking that into consideration, Gama Vaz (1993) observed that 7.5% of urinary schistosomiasis patients in Mozambique develop bladder carcinoma, and 59% of them developed squamous cells’ carcinoma [68]. In 1994, Taquinho et al. (1994) conducted a study with 1240 participants from one to 70 years old in Boane, Maputo province, and observed that 40.7% had urinary schistosomiasis, and 74.5% of infected people were between 5 and 19 years old. In 1998, a study of 434 schoolchildren from 6 to 16 years old randomly selected from random selected schools of the city in Maputo showed a lower overall prevalence of urinary schistosomiasis (11.3%) [69], nevertheless only a sample from each participant was evaluated. In 2005–2007, Augusto et al. (2009) updated the national data available on Schistosomiasis. They observed 47% for *S. haematobium* and 1% *S. mansoni* overall. The prevalence of *S. haematobium* increased slightly with age, reaching a peak in the schoolchildren 10–14 years (47.4%) and 45.3% in children > 14 years of age. The highest *S. haematobium* prevalence was in the northern provinces (Cabo Delgado, Niassa, Nampula, and Zambezia) and in certain provincial capital cities. The lowest *S. haematobium* prevalences were in Inhambane (19.9%), Gaza (21.4%) and Tete Province (33.4%) [70]. By 2012, Mozambique was considered highly endemic for schistosomiasis (> 50%), with Ghana, Liberia and Sierra Leone [71]. Indeed, the last cross-sectional study in northern Mozambique, in 2014, still reflected a prevalence of urinary schistosomiasis of 59.1% [72]. The diagnostic method used was not described in some of the reviewed studies. In those in which it is described, a wide variety of diagnostic methods is used, including urine filtration for *S. haematobium* and different microscopy techniques such as Ritchie and Kato-Katz techniques for *S. mansoni* detection.

##### Soil-transmitted helminthiasis

The first data available on soil-transmitted helminths (STH) infections appears in 1958. De Morais (1958) [73] conducted a cross-sectional study in Inhaca island, Maputo, with 80 people aged from 3 to 30 years old and they found very high prevalences for *Ascaris lumbricoides* and *Trichuris trichiura* (86.3 and 52.5% respectively). A latter cross-sectional study was performed in Maputo district in 1961 and out of 20 Inhaca participants showed that 18 of them were infected with *A. lumbricoides*, five with *Ancylostoma duodenale* and one with *Strongyloides stercoralis* infection. STH prevalence observed in the complete study in Maputo district in 1961 (420 participants) was also headed by *A. lumbricoides* (57.61%), followed by *A. duodenale* (16.4%) and *S. stercoralis* (1.2%) [74]. In Maputo city, a cross-sectional study before conducting an evaluation of a sanitation programme in 97 households (244 people) in 1987 showed 23% of STH infection with a higher infection in 5 to 15 years old (35%) [75]. Enosse et al. (1995) [76] conducted household feaces collection in 30 clusters of households from Infulene valley (Maputo city rural area) and Maholas (Maputo city semi-urban area) and detected STH prevalence still headed by *A. lumbricoides* (17.1%), followed by *T. trichuria* (15.4%), *A. duodenale* (6.4%) and *S. stercoralis* (1.7%). However, the STH with highest prevalence in Maputo Central Hospital in children between 1.5 and 48.2 months of age in 2009 was *T. trichuria* (6.5%), while the prevalence of *A. lumbricoides* was only 2.2% [77]. Three studies were conducted in the city of Beira. The first one, in 2004, disclosed that helminths infections were highly prevalent in the suburban Beira inhabitants of all ages. From 497 people, 95.5% in children aged 2–6 years were infected by STH; 97% in aged 7 to 15 and 76.4% in aged > 15 years old. Concretely, *T. trichuria* was the most common parasite, whereas *A. lumbricoides* was less common and hookworms were rare [78]. The second study examined all samples by direct smear, formal-ether concentration (FEC), Kato-Katz smear, Baermann method, coproculture and real-time PCR and found that 96% of the participants (*n* = 303) from an informal settlement, Inhamudima, were infected by at least one STH in 2007, and almost half of them by three STH or more. *T. trichuria* was the most abundant (93%), followed by *A. lumbricoides* (56%), *S. stercoralis* (48%) and hookworm (38%). The third study, conducted in 2013, detected lower specific prevalences by STH ranging between 35 and 53% [79]. In the northern provinces of Mozambique, three cross-sectional studies were performed. Firstly, in contrast to Maputo and Beira, Pinhão (1965) [80] observed the highest prevalence for *A. duodenale* (12%), and lower for *A. lumbricoides* (3–5%) and *S. stercoralis* (0–2%) in Tete. In addition, Casmo et al. (2014) [72] during 2005–2007 detected 31.3% of hookworm prevalence in Cabo Delgado, Niassa and Nampula. Moreover, a survey in 3 Nampula schools in 2009, *A. duodenale* was also the STH with main prevalence (18.9%) [81]. All prevalences were determined by different microscopy techniques; while most used Ritchie and Willies techniques, only the study conducted by Meurs et al. (2017) [82] in Inhamudina used the Baermann technique to detect *S. stercoralis.*

No articles or reports meeting our criteria were found for buruli ulcer, yaws, leishmaniasis, fascioliasis and echinococcosis.

### Discussion

Mozambique has been reported to be endemic for 11 NTDs since 1950. Every NTD has had their own profile in the country: while some are widely spread (e.g. soil-transmitted helminths), others are only localized in some areas of the country (e.g. HAT). Northern provinces (Cabo Delgado, Nampula, Niassa and Tete), the north-central province (Zambezia) and Maputo province and city are the regions with more NTDs’ evidence and might be the most affected areas. Ecological and demographic conditions could partly explain that. The north has higher rainfall and higher temperatures resulting in a sub-humid area and the center has higher number of floods, whereas the south is more arid [83]. Moreover, north and center are the areas with highest poverty indices and highest proportion of usage of unsafe water and sanitation [84]. Furthermore, Nampula, Zambezia and Maputo are the provinces with highest population density [85].

**Box 1:** NTDs’ research and control programme gaps in Mozambique that could enhance national NTDs programs

Mozambique NTDs’ research and control needs - To enhance NTDs’ surveillance and to use prediction mapping in Mozambique to describe NTDs’ presence and distribution around the country. - To develop and to utilize simpler field testing tools. - To integrate national NTDs programs’ strategies, such as mass drug administration and vector control. - To create a trans-boundering alliance for NTDs programs among neighbouring countries. - To incorporate a one health approach.	

However, data are missing on both space and time scales. A few regions do not hold data for some NTDs, which does not imply no NTDs transmission. A possible explanation is that resources to generate evidence are allocated to either higher risk regions for NTDs or regions with more technical facilities [2], for instance studies based in Maputo city or Maputo Central Hospital. Fortunately, the national program is running specific programs for LF, schistosomiasis and STH at a district level that will provide new data on prevalence and distribution soon. However, for those diseases with no current national program, their epidemiological status is unknown and the reliable epidemiological data is limited. It is the case of cysticercosis or onchocerciasis, which have no data available since 2007 even though both are considered endemic by WHO [86]. Some NTDs, such as echinococcosis, have never been evaluated in Mozambican territory. Along these lines, we also observed that during the period of the Mozambican War of Independence the number of publications declined. After this event, data on some diseases was missing for several years: during 20 years for HAT and 30 years for trachoma, until later studies showed that they were still present.

Moreover, national and international disease priorities also affect surveillance and research resources allocation. Many NTDs have a regional relevance but are not widely distributed [3]. Considering that low-income countries usually have restricted resources for health programs [2], the resilient epidemic of HIV/AIDS, tuberculosis and malaria in sub-Saharan Africa could overcome NTDs rank [87, 88], .In fact, it has been observed that these diseases dominate infectious diseases research whereas global NTDs research intensity is still low [88, 89].

On the other hand, the use of diverse diagnostic techniques for the same disease makes the data difficult to harmonize and compare among the studies across space and time. Studies were conducted using tests with different sensitivities, especially for infections detected by microscopy in feces samples, such as *S. mansoni* and STH. In particular, only one study used Baermann technique for *S. stercoralis* identification, the most sensitive microscopy test nowadays for this parasite [90]. In addition, some surveys identified the NTD as a secondary effect of the main study endpoint, thus, the epidemiological parameter could be biased by report or measurement.

Furthermore, this review has their own limitations. From 306 records identified, 17% could not be acquired neither online nor on paper. Moreover, some studies targeting NTDs have to be discarded because the sample size and study design was unclearly defined. Thus, the remaining records were few. In addition, since data was from different areas of the country, several years apart and diagnosed with different sensitivity tests, to conduct a meta-analysis was unfeasible. However, the NTDs epidemiological records assembled for the first time in the results of this review could guide the future directions of NTDs research and health programs.

Enhanced surveillance in Mozambique is needed to evaluate the presence and distribution of the diverse NTDs in the country, to assess possible increase or re-emergence of NTD infection in the country, and to inform policy makers to target control and elimination strategies. For a reliable surveillance, to map risk areas where NTD data is unavailable is essential, especially if elimination is not certified. We recommend the use of remote sensing predictors of disease ecology and accurate mapping to tackle NTDs hotspots, primarily after the last natural disasters recently occurred in the country. In addition, we prompt the need to develop and use simpler field test tools, especially for *S. stercoralis*. On top of that, we also support the integration of NTDs program in one when possible (e.g. MDA or vector control), the partnership among national health programs across countries to control trans-boundering diseases (e.g for HAT), and the integration of animal health – the One Health approach. This could diminish logistical and financial effort, provide data for populations difficult to access and improve NTDs’ surveillance [2, 9].

Higher surveillance in NTDs would improve people’s life. But it would not only decrease NTDs infections, it would also diminish other health complications, such as HIV, epilepsy or cancer. As example, genital schistosomiasis has been observed to quadruple the chances of HIV infection [91], neurocisticercosis is associated with 30% of the global epilepsy [92], and *S. haematobium* control has been associated with bladder cancer decline [93]. Hence, identifying its infection and reducing transmission could enhance other population health conditions and reduce national health spending.

### Conclusions

This manuscript reviews the known prevalence and distribution of the most relevant NTDs in Mozambique since 1950 until 2018. All NTDs had very different profile on distribution and data availability along the period and across the country. This review provides key elements to progress towards the control and interruption of transmission of these diseases in the country, as a key contributor to achieving the SGDs and ultimately improving life of millions of people at risk.

## Supplementary information

**Additional file 1.** Multilingual abstracts in the five official working languages of the United Nations.

**Additional file 2.** PRISMA checklist.

**Additional file 3.** PRISMA Flow Diagram.

**Additional file 4.** Search terms used to conduct electronic literature search.

**Additional file 5.** List of manuscripts included in Fig. 3

**Additional file 6.** List of manuscripts included in the systematic review.

## Data Availability

All data analysed during this study are included in this published article in Additional file 6.

## Abbreviations

AIDS: Acquired Immunodeficiency Syndrome
CF: Complement fixation
CSF: Cerebrospinal fluid
DENV: Dengue virus
ELISA: Enzyme-linked immunosorbent assay
FAT: Fluorescent antibody test
FEC: Formal-ether concentration
HAT: Human African trypanosomiasis
HI: Hemagglutination-inhibition
LF: Lymphatic filariasis
MCT: Mission to Combat Trypanossomiasis
NTDs: Neglected tropical diseases
PCR: Polymerase chain reaction
PRISMA: Preferred Reporting Items for Systematic Reviews and Meta-Analysis
REMO: Rapid Epidemiological Mapping of Onchocerciasis
SDG: Sustainable Development Goal
STH: Soil-transmitted helminths
WHO: World Health Organization
