# Supplementary material for: Frequency and distribution of neglected tropical diseases in Mozambique: a systematic review
Source: Infect Dis Poverty. 2019 Dec 13;8:103. doi: 10.1186/s40249-019-0613-x (PMC6909500; doi:10.1186/s40249-019-0613-x)
Supplement: Supplementary file 1 — Additional file 1. Multilingual abstracts in the five official working languages of the United Nations. [file 40249_2019_613_MOESM1_ESM.pdf]

Translation of the abstract into the five official working languages of the United Nations

تواتر وتوزع الأمراض المدارية المُهمَلة في موزامبيق: مراجعة منهجية

بيرتا غراو-بوجول، ماريليا ماسانغاي، خورخي كانو، كارمن ماروتو، الكانتارا نديف، فرانسيسكو ساوتي، خوسيه ميونوز

نبذة مختصرة

الخلفية: تؤثر أمراض المناطق المدارية المهملة على أكثر من مليار شخص يعيشون في ظروف معرضة للخطر. على الرغم من المبادرات العامة والخاصة التي ساهمت مؤخرًا في سد ثغرات الأمراض المنقولة بالاتصال الجنسي على الانتشار والتوزيع على الصعيدين الوطني والمحلي، لا تزال هناك حاجة إلى مزيد من البيانات الوبائية من أجل تدخلات مكافحة وإزالة فعالة.

النص الرئيسي: تعتبر موزامبيق واحده من البلدان التي لديها أعلى عبء من حيث أمراض المناطق المدارية المُهمَلة على الرغم من ندره البيانات المتاحة. تهدف هذه الدراسة إلى إجراء مراجعة منهجية للبيانات المنشورة المتاحة حول عبء وتوزع أمراض المناطق المدارية المهملة المختلفة في موزامبيق منذ كانون الثاني/يناير 1950 حتى كانون الأول/ديسمبر 2018. حددنا المخطوطات باستخدام قواعد البيانات الإلكترونية (إطار نظام البحث على الشبكة العالمية (Pubmed)، وإم بيس (EmBase) والصحة العالمية) والمطبوعات الورقية والمنشورات غير الرسمية من وزاره الصحة في موزمبيق. وكانت المخطوطات مستوفية لمعايير الإدراج وهي: دراسات مقطعية، ودراسات بيئية، وفئات، وأتراب، ومراجعات منهجية، ومراجعات سردية تُسجل المعلومات الوبائية للأمراض المستوطنة في موزامبيق. تم استبعاد دراسات مراقبة الحالات، والرسائل إلى المحرر، وتقارير الحالة، وسلاسلا الحالات من الحالات المستوردة. وتم تحديد ما مجموعه 466 مخطوطًا في البداية وتم تضمين 98 منها أخيرًا بعد المراجعة باتباع إرشادات بريسما (PRISMA). وتم الإبلاغ عن أحد عشر حالة من أمراض المناطق المدارية المهملة في موزامبيق خلال فتره الدراسة. واكتشف في المقاطعات الشمالية (نامبولا، وكابو دلغادو، ونياسا، وتيتي، وزامبيزيا) ومقاطعة مابوتو أكبر عدد من حالات أمراض المناطق المدارية المهملة. كان لكل مرض بياناته الخاصة: في حين تم الإبلاغ عن داء البلهارسيا بشكل مستمر منذ 1952 وحتى يومنا هذا، فإن آخر البيانات المتاحة عن داء كلابية الذنب وداء الكيسات المذنبة كان منذ عام 2007 ولم يتم تقييم داء المشوكات السنخي أبداً في البلاد. وهكذا، تم تحديد كل من الفجوات في المكان والزمان على وبائيات أمراض المناطق المدارية المهملة.

الاستنتاجات: تجمع هذه المراجعة عبء وتوزيع أمراض المناطق المدارية المهملة في موزامبيق، وتسهم في فهم وبائيات أمراض المناطق المدارية المهملة في البلد وتسلط الضوء على فجوات المعرفة. وبالتالي، توفر الدراسة عناصر أساسية للتقدم نحو السيطرة ووقف انتقال هذه الأمراض في البلاد.

Translated from English version into Arabic by Sami Al Jaouni, Revised by Muhannad Albayk Jaam, through

TRANSLATORS
WITHOUT BORDERS

莫桑比克被忽视热带病的发病频率和分布：系统综述

Berta Grau-Pujol, Marilia Massangaie, Jorge Cano, Carmen Maroto, Alcino Ndeve, Francisco Saute and Jose Muñoz

摘要

引言：被忽视热带病影响着 10 多亿贫困人口。尽管最近公共和私营部门已采取行动致力于

填补被忽视热带病在国家和地区层面的流行和分布空白，但还需要更多的流行病学数据以便采取有效的控制和消除措施。
正文：虽然缺乏可靠数据，但莫桑比克被认为是被忽视热带病负担最严重的国家之一。本研究收集了 1950 年 1 月至 2018 年 12 月期间已发表的关于莫桑比克不同被忽视热带病的负担和分布的可靠数据，并对其进行系统性回顾分析。搜索了电子数据库(Pubmed、EmBase 和 Global Health)、纸质出版物和莫桑比克卫生部的灰色文献。符合纳入标准的数据包括：横断面研究、生态学研究、队列研究、报告、系统综述和描述莫桑比克被忽视热带病流行病学信息的叙述性综述。排除了病例对照研究、通讯类文章、病例报告和输入病例被排除在外。符合以上标准的有 466 份数据，遵循 PRISMA 指南进行修订后最终纳入 98 份。在研究年限中，莫桑比克报告了 11 例被忽视热带病。北部省份(楠普拉、德尔加杜角、尼亚萨、太特和赞比西亚省)和马普托省诊断出的被忽视热带病病例数较高。每一种疾病的情况不同：自 1952 年至今一直有血吸虫病的报告，但直至 2007 年才有盘尾丝虫病和囊尾蚴病的科学数据。未对包虫病进行过评估。因此，被忽视热带病流行病学上的空间和时间空白已被确定。
结论：本综述汇总了莫桑比克被忽视热带病的负担和分布情况，有助于了解莫桑比克被忽视热带病的流行病学现状，并强调了需进一步研究之处。因此，该研究为在该国控制和阻断这些疾病的传播方面提供了关键科学依据。

Translated from English version into Chinese by Peng Song, edited by Jin Chen

CHINESE CENTER FOR DISEASE CONTROL AND PREVENTION
NATIONAL INSTITUTE OF PARASITIC DISEASES

Fréquence et distribution des maladies tropicales négligées au Mozambique : une revue systématique

Berta Grau-Pujol, Marilia Massangaie, Jorge Cano, Carmen Maroto, Alcino Ndeve, Francisco Saute et Jose Muñoz

Résumé

Contexte : Les maladies tropicales négligées (NTD) touchent plus d'un milliard de personnes vivant dans des conditions de vulnérabilité. En dépit d'initiatives publiques et privées contribuant récemment à combler les lacunes des MTN en matière de prévalence et de distribution au niveau national et local, des données épidémiologiques supplémentaires sont encore nécessaires pour des interventions de contrôle et d'élimination efficaces.

Texte principal : Le Mozambique est considéré comme l'un des pays où la charge des NTD est la plus importante, bien que les données disponibles soient peu nombreuses. Le but de cette étude est de mener une revue systématique des données publiées disponibles en ce qui concerne la charge et la distribution des différentes NTD à travers le Mozambique de janvier 1950 à décembre 2018. Nous avons identifié les manuscrits à l'aide de bases de données électroniques (Pubmed, EmBase, et Global Health), de publications papier, et de littérature grise du Ministère de la Santé mozambicain. Les manuscrits correspondant aux critères d'inclusions étaient : les analyses transversales, les études écologiques, les cohortes, les rapports, les revues systématiques, et les revues narratives capturant les informations épidémiologiques des NTD endémiques au Mozambique. Les études cas-témoins, les lettres aux éditeurs, les rapports de cas et les séries de cas concernant des cas importés ont été

exclus. Un total de 446 manuscrits a été identifié pour commencer, et 98 ont finalement été inclus une fois la révision d'après les lignes directrices PRISMA effectuée. Onze cas de NTD ont été signalés au Mozambique pour la période étudiée. Les provinces du nord (Nampula, Cabo Delgado, Niassa, Tete et Zambezia) ainsi que la province de Maputo présentaient le plus grand nombre de NTD détectées. Chaque maladie avait son profil de rapport propre : tandis que la schistosomiase est signalée en continu depuis 1952, les dernières données disponibles pour l'onchocercose et la cysticercose datent de 2007, et l'échinococcose n'a jamais été évaluée dans le pays. Ainsi, les lacunes temporelles et géographiques concernant l'épidémiologie des NTD ont été identifiées.
Conclusions : Cette revue assemble la charge et la distribution des NTD au Mozambique, contribue à la compréhension de l'épidémiologie des NTD au Mozambique, et à mettre en avant les lacunes au niveau des connaissances. L'étude fournit donc des éléments clés pour avancer vers le contrôle et l'interruption de la transmission de ces maladies dans le pays.

Translated from English version into French by LAURIE MOURET, Revised by Emilie Rigault Fourcadier, through

Диагностика и лечение забытых тропических болезней.

Берта Грау-Пухоль, Марилия Массанге, Хорхе Кано, Кармен Марото, Альсино Ндеве, Франсиско Соте и Хосе Муньос

Краткое описание

Справочная информация: Забытые тропические болезни (ЗТБ) поражают более одного миллиарда людей, живущих в уязвимых условиях. Несмотря на общественные и частные инициативы, которые в последнее время способствуют заполнению пробелов в контроле распространения ЗТБ на национальном и местном уровнях, для эффективного вмешательства и мер по ликвидации все еще необходимы дополнительные эпидемиологические данные.
Основной текст: Мозамбик считается одной из стран с самым высоким бременем ЗТБ, хотя доступных данных мало. Это исследование направлено на проведение систематического обзора опубликованных доступных данных о бремени и распространении различных ЗТБ по Мозамбику с января 1950 года по декабрь 2018 года. Мы определили рукописи с использованием электронных баз данных (Pubmed, EmBase и Global Health) и бумажных публикаций и малоизвестной литературы из Министерства здравоохранения Мозамбика. К рукописям, отвечающим критериям включения, относятся: перекрестные исследования, экологические исследования, когорты, отчеты, систематические и описательные обзоры, содержащие эпидемиологическую информацию об эндемических ЗТБ в Мозамбике. Были исключены тематические исследования, письма редактору, отчеты по конкретным случаям и ряд импортированных случаев. Изначально было идентифицировано 466 рукописей, и, наконец, 98 были включены после пересмотра в соответствии с рекомендациями PRISMA.

Во время исследований выявлено 2 случая нежелательных явлений. В северных провинциях (Нампула, Кабо-Дельгадо, Ниасса, Тете и Замбезия) и в провинции Мапуту было выявлено более высокое количество ЗТБ. Каждое заболевание имеет свой собственный отчетный профиль: если шистосомоз постоянно регистрируется с 1952 года до настоящего времени, то онхоцеркоз и цистицеркоз - с 2007 года, а эхинококкоз в стране никогда не подвергался оценке. Таким образом, были выявлены пробелы в эпидемиологии ЗТБ как в пространстве, так и во времени.
Выводы: В этом обзоре собраны данные о бремени и распространении ЗТБ в Мозамбике, а также внесены вклад в понимание эпидемиологии ЗТБ в Мозамбике и выявлены пробелы в знаниях. Следовательно, исследование предоставляет ключевые элементы для продвижения к контролю и прекращению передачи этих заболеваний в стране.

Translated from English version into Russian by Maria Petrenko, Revised by Michael Orlov, through

TRANSLATORS
WITHOUT BORDERS

Frecuencia y distribución de las enfermedades tropicales desatendidas en Mozambique: una revisión sistemática

Berta Grau-Pujol, Marilia Massangaie, Jorge Cano, Carmen Maroto, Alcino Ndeve, Francisco Saute y Jose Muñoz

Resumen

Introducción: las enfermedades tropicales desatendidas (las ETD) afectan a más de mil millones de personas en condiciones vulnerables. A pesar de que las iniciativas públicas y privadas están contribuyendo recientemente para subsanar las lagunas en torno a la distribución y la prevalencia nacional y local de las ETD, se necesitan más datos epidemiológicos para conseguir un control eficaz e intervenciones para su eliminación.
Texto principal: Mozambique es considerado como uno de los países con mayor carga de ETD, aunque hay pocos datos disponibles al respecto. Este estudio busca llevar a cabo una revisión sistemática de los datos publicados sobre la carga y la distribución de distintas ETD en todo Mozambique desde enero de 1950 hasta diciembre de 2018. Hemos identificado manuscritos mediante bases de datos electrónicas (Pubmed, EmBase y Global Health) y publicaciones, y literatura gris del Ministerio de Salud de Mozambique. Los manuscritos que cumplían los criterios de inclusión consistieron en: estudios transversales, estudios ecológicos, cohortes, informes, revisiones sistemáticas y revisiones narrativas que recogen la información epidemiológica de las ETD endémicas de Mozambique. Se excluyeron estudios de casos y controles, cartas al editor, estudios de casos y series de casos. Inicialmente se identificaron 466 manuscritos y finalmente, después de la revisión siguiendo la declaración PRISMA, se incluyeron 98. A lo largo de la investigación se notificaron once ETD en Mozambique. Las provincias del norte (Nampula, Cabo

Delgado, Tete y Zambezia) y Maputo presentan el número más alto de ETD. Cada enfermedad tiene su propio informe: mientras que se han notificado esquistosomiasis de forma continua desde 1952 hasta la actualidad, los últimos datos que se conocen de la oncocercosis y de la cisticercosis son de 2007 y nunca se ha evaluado la equinococosis en el país. Por tanto, se han identificado deficiencias espaciales y temporales en la epidemiología de las ETD.

Conclusiones: esta reseña recopila la carga y la distribución de las ETD en Mozambique, y contribuye a la comprensión de la epidemiología de las ETD en Mozambique y destaca deficiencias en el conocimiento. Por lo tanto, esta investigación proporciona elementos claves para progresar en el control y en la interrupción de la transmisión de estas enfermedades en el país.

Translated from English version into Spanish by Verónica Olmos González, Revised by Macarena Nieva, through

TRANSLATORS
WITHOUT BORDERS
