# Supplementary material for: Frequency and distribution of neglected tropical diseases in Mozambique: a systematic review
Source: Infect Dis Poverty. 2019 Dec 13;8:103. doi: 10.1186/s40249-019-0613-x (PMC6909500; doi:10.1186/s40249-019-0613-x)
Supplement: Supplementary file 3 — Additional file 3. PRISMA Flow Diagram. [file 40249_2019_613_MOESM3_ESM.doc]

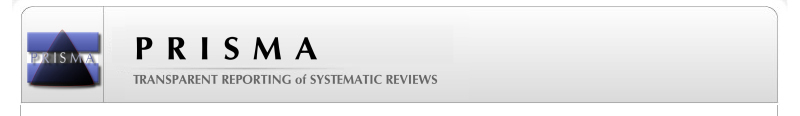
**PRISMA 2009 Flow Diagram**

**Screening**

**Included**

**Eligibility**

**Identification**

Records identified through database searching
(n = 431 )

Additional records identified through other sources
(n = 35 )

Records after duplicates removed
(n = 306 )

Records screened
(n = 255 )

Records excluded
(n = 126 )

Full-text articles assessed for eligibility
(n = 129 )

Full-text articles excluded, with reasons
(n = 31 )

Studies included in qualitative synthesis
(n = 98 )

Studies included in quantitative synthesis (meta-analysis)
(n = NA )
