# Supplementary material for: Frequency and distribution of neglected tropical diseases in Mozambique: a systematic review
Source: Infect Dis Poverty. 2019 Dec 13;8:103. doi: 10.1186/s40249-019-0613-x (PMC6909500; doi:10.1186/s40249-019-0613-x)
Supplement: Supplementary file 4 — Additional file 4. Search terms used to conduct electronic literature search. [file 40249_2019_613_MOESM4_ESM.docx]

**Additional file 4. Search terms used to conduct electronic literature search.**

(nematode* OR helmint* OR soil transmitted helmint* OR STH* OR ascari* OR roundworm* OR trichur* OR whipworm* OR hookworm* OR necator OR ancylostom* OR strongy* OR enterobi* OR geohelmint* OR schisto* OR haematobium OR mansoni OR bilharzia* OR esquisto* OR lymphatic filaria* OR LF OR elephantiasis OR bancrofti OR onchocerc* OR oncocerc* OR river blindness OR simulium OR blackfl* OR guinea worm* OR dracuncul* OR fluke* OR cysticerc* OR taenia* OR tenia* OR solium OR saginata OR tapeworm* OR fasciola* OR echinococ* OR equinococ* OR hydatid* OR hidatidosis OR leishmania* OR infantum OR donovani OR trofica OR sand fl* OR sandfl* OR african trypano* OR trypano* OR HAT OR brucei OR gambiense* OR rhodesiense* OR sleeping sickness OR tsetse OR glossina OR trachoma* OR tracoma* OR trichiasis OR buruli ulcer* OR lepro* OR lepra* OR Hansen OR rabies OR lyssavir* OR RABV OR dengue OR aedes OR albopictus OR aegypti) AND (epidemiology OR incidence OR prevalence) AND Mozambique.
