# Supplementary material for: Frequency and distribution of neglected tropical diseases in Mozambique: a systematic review
Source: Infect Dis Poverty. 2019 Dec 13;8:103. doi: 10.1186/s40249-019-0613-x (PMC6909500; doi:10.1186/s40249-019-0613-x)
Supplement: Supplementary file 5 — Additional file 5. List of manuscripts included in Fig. 3 [file 40249_2019_613_MOESM5_ESM.docx]

**Additional file 5. List of manuscripts included in Figure 3.**

| **Neglected Tropical disease** | **Publication** | **Location of the data** | **Year of the data** | **Journal** |
| --- | --- | --- | --- | --- |
| Dengue | Massangaie et al (2016) | Pemba and Nampula | 2014 | The American Journal of Tropical Medicine and Hygiene |
|  | Mugabe et al (2018) | Quelimane | 2014-2016 | Plos One |
|  | Muianga et al 2018) | Pemba | 2014 | Vector-borne and zoonotic diseases |
| Lymphatic filariasis | Manhenje et al (2013) | Niassa, Cabo Delgado, Nampula, Zambezia | 2008 | Geospatial Health |
| Rabies | WHO (2013) | Mozambique | 2011 | Report WHO |
|  | Salomao et al (2017) | Maputo and Matola | 2014 | Plos Neglected Tropical Diseases |
| Schistosomiasis | Schur et al (2012) | Mozambique | 2010 | Plos Neglected Tropical Diseases |
|  | Schur et al (2013) | Mozambique | 2010 | Acta Tropica |
|  | Casmo et al (2014) | Northern Mozambique (Nampula, Cabo Delgado and Niassa) | 2014 | Revista do Instituto de Medicina Tropical de São Paulo |
|  | Ezeamama et al (2016) | Mozambique | 2009 | BMC Infectious Diseases |
|  | Phillips et al (2017) | Cabo Delgado | 2011-2015 | Plos Neglected Tropical Diseases |
|  | Phillips et al (2018) | Cabo Delgado | 2011 | Parasites & Vectors |
| Soil-transmitted helminths | Guidetti et al (2011) | Marrere, Nampula | 2009 | Le Infezioni in Medicina |
|  | Fonseca et al (2014) | Maputo | 2009 | The Journal of Infection in Developing Countries |
|  | Kaisar et al (2013) | Beira | 2013 | Tropical Medicine and International Health |
| Trachoma | Abdala et al (2017) | Mozambique | 2012-2015 | Ophthalmic Epidemiology |
