# Supplementary material for: Frequency and distribution of neglected tropical diseases in Mozambique: a systematic review
Source: Infect Dis Poverty. 2019 Dec 13;8:103. doi: 10.1186/s40249-019-0613-x (PMC6909500; doi:10.1186/s40249-019-0613-x)
Supplement: Supplementary file 6 — Additional file 6. List of manuscripts included in the systematic review. [file 40249_2019_613_MOESM6_ESM.docx]

**Additional file 6. List of manuscripts included in the systematic review.**

| **Neglected Tropical disease** | **Publication** | **Location of the data** | **Year of the data** |
| --- | --- | --- | --- |
| Buruli ulcer | - | - | - |
| Cystcercosis | Vilhena et al (1990) | Tete city | 1990 |
|  | Vilhena et al (1999) | Maputo | 1993 |
|  | Mafojane et al (2003) | Tete city, Tete province, Maputo | 1994, 2002 |
|  | Noormahomed et al (2003) | Maputo | 1998 |
|  | Phiri et al (2003) | Tete, Maputo | 1994, 1999 |
|  | Assane (2009) | Tete | 2007 |
|  | Afonso et al (2011) | Maputo | 1968, 2003 |
|  | Trevisam et al (2013) | Agonia district, Tete. | 2007 |
|  | Assane et al (2015) | Agonia district, Tete. | 2007 |
|  | Trevisan et al (2018) | Agonia district, Tete. | 2007 |
| Dengue | Gubler et al (1986) | Pemba | 1984-1985 |
|  | Miyar et al (1987) | Pemba | 1984-1985 |
|  | Abreu et al. (1987) | Pemba | 1985 |
|  | Massangaie et al (2016) | Pemba and Nampula | 2014 |
|  | Gudo et al (2018) | Mozambique | 1984-1985 |
|  | Mugabe et al (2018) | Quelimane | 2014-2016 |
|  | Muianga et al 2018) | Pemba | 2014 |
| Dracunculiasis | World Health Organization (2007) | Mozambique | 2007 |
| Echinoccocosis | - | - | - |
| Fasciolasis | - | - | - |
| Human African Trypanosomiais | Urban et al (1987) | Niassa, Tete, Cabo Delgado. | 1975-1984 |
|  | Urban et al (1987) | Tete | 1979-1984 |
|  | Davies et al (1983) | Mozambique | 1940-1982 |
|  | Neves et al (1971) | Mozambique | 1950-1970 |
|  | World Health Organization (1960) | Mozambique | 1943-1960 |
|  | Ministerio de Saúde de Mozambique (1958) | Mozambique | 1958 |
|  | Ministerio de Saúde de Mozambique (1957) | Mozambique | 1957 |
|  | Ministerio de Saúde de Mozambique (1956) | Mozambique | 1956 |
|  | Ministerio de Saúde de Mozambique (1954) | Mozambique | 1954 |
|  | Ministerio de Saúde de Mozambique (1953) | Mozambique | 1953 |
|  | de Andrade Silva (1952) | Niassa | 1931-1940 |
|  | Ministerio de Saúde de Mozambique (1950) | Mozambique | 1950 |
|  | Simarro et al (2010) | Mozambique | 2002, 2004 |
|  | Franco et al (2017) | Mozambique | 2002, 2004 |
| Leishmaniasis | - | - | - |
| Leprosy | Phaff et al (2003) | Northern Mozambique | 1996-2002 |
|  | Stuip et al (2003) | Niassa | 1999 |
|  | WHO (2005) | Mozambique | 2003 |
|  | WHO (2005) | Mozambique | 1993, 2002-2004 |
|  | WHO (2006) | Mozambique | 2005 |
|  | WHO (2007) | Mozambique | 2006 |
|  | WHO (2007) | Mozambique | 2007 |
|  | WHO (2008) | Mozambique | 2008 |
|  | WHO (2009) | Mozambique | 2009 |
|  | WHO (2010) | Mozambique | 2010 |
|  | WHO (2011) | Mozambique | 2011 |
|  | WHO (2012) | Mozambique | 2012 |
|  | Kruijff (2015) | Mozambique | 2008-2013 |
|  | WHO(2017) | Mozambique | 2008-2012, 2015-2017) |
| Lymphatic filariasis | Santos Reis (1955) | MCT area, Nampula | 1953 |
|  | Pinhao (1961) | Zambeze valey, Sofala and Zambezia | 1959-1960 |
|  | de Azevedo (1964) | Northern Mozambique (Zambezia, Tete, Manica, Sofala, Cabo Delgado) | 1959-1969 |
|  | Lourenço et al (1990) | Pemba | 1990 |
|  | Gujral et al (2003) | Cabo Delgado | 1995-2000 |
|  | Manhenje et al (2013) | Niassa, Cabo Delgado, Nampula, Zambezia | 2008 |
| Onchocerciasis | MISAU/WHO (1996) | Zambezia and Tete | 1996 |
|  | Barreto et al (1998) | Cabo Delgado, Niassa, Tete and Zambezia. | 1998 |
|  | Noormahomed (1998) | Niassa and Zambezia | 1998 |
|  | Noormahomed et al (2016) | Niassa, Cabo Delgado, Nampula, Zambezia, Tete, Manica, Sofala and Inhambane | 2001, 2007 |
| Rabies | Dias et al (1987) | Mozambique, all provinces | 1978-1982 |
|  | WHO (2013) | Mozambique | 2011 |
|  | Salomao et al (2017) | Maputo and Matola | 2014 |
| Schistosomiasis | Alves (1957) | Mozambique | 1952-1956 |
|  | De Morais (1959) | Mozambique | 1956 |
|  | Lourenço Marques, Institut of Medical Research (1959) | Mozambique | 1956 |
|  | Ferreira (1963) | Periferia Maputo: Bela vista, Catembe, Inhaca, Manhoca, Catuane. | 1961 |
|  | McMullen et al (1962) | Mozambique | 1962 |
|  | Coutinho (1964) | Niassa | 1964 |
|  | Rey (1978) | Mozambique | 1957, 1961, 1976-1978 |
|  | Paulo et al (1984) | Mueda, Northern Mozambique | 1984 |
|  | WHO (1987) | Mozambique | beginning- 1987 |
|  | Rey et al (1987) | Village in Marracuene district | 1961-1981 |
|  | Gama (1993) | Mozambique | 1993 |
|  | Gama (1993) | Maputo | 1985, 1991, 1993 |
|  | Traquinho et al (1994) | Maputo province | 1992, 1993 |
|  | Traquinho et al (1998) | Cabo Delgado | 1987, 1998 |
|  | Enosse at al (1995) | Infulene valley (rural Maputo) and Maholas (semi-urban Maputo) | 1995 |
|  | Gujral et al (2000) | Maputo | 1996 |
|  | Antunes et al (2009) | Suburban Beira | 2004 |
|  | Augusto et al (2009) | Matola and Maputo | 2004 |
|  | Augusto et al (2009) | Mozambique | 2005-2007 |
|  | Schur et al (2012) | Mozambique | 2010 |
|  | Schur et al (2013) | Mozambique | 2010 |
|  | Casmo et al (2014) | Northern Mozambique (Nampula, Cabo Delgado and Niassa) | 2014 |
|  | Ezeamama et al (2016) | Mozambique | 2009 |
|  | Phillips et al (2017) | Cabo Delgado | 2011-2015 |
|  | Phillips et al (2018) | Cabo Delgado | 2011 |
| Soil-transmitted helminths | De Morais (1958) | Inhaca island | 1958 |
|  | Ferreira (1963) | Maputo district: Bela vista, Catembe, Inhaca, Manhoca, Catuane. | 1961 |
|  | Pinhao (1965) | Tete | 1961 |
|  | Muller et al (1989) | Maputo | 1987 |
|  | Enosse at al (1995) | Infulene valley (rural Maputo) and Maholas (semi-urban Maputo) | 1995 |
|  | Antunes et al (2009) | Suburban Beira | 2004 |
|  | Augusto et al (2009) | Mozambique | 2005-2007 |
|  | Casmo et al (2014) | Nampula, Cabo Delgado and Niassa | 2005-2007 |
|  | Guidetti et al (2011) | Marrere, Nampula | 2009 |
|  | Fonseca et al (2014) | Maputo | 2009 |
|  | Kaisar et al (2013) | Beira | 2013 |
|  | Meurs et al (2017) | Suburban Beira | 2007 |
| Trachoma | Cairncross et al (1987) | 2 villages in Mueda, Northern Mozambique | 1982, 1983 |
|  | Abdala et al (2017) | Mozambique | 2012-2015 |
|  | Paulo et al (1984) | Mueda, Northern Mozambique | 1984 |
| Yaws | - | - | - |
